# Efficacy of Bilateral Lower-Limb Training Over Unilateral Lower-Limb Training To Reeducate Balance and Walking in Post-Stroke Survivors: A Randomized Clinical Trial

**DOI:** 10.7759/cureus.30748

**Published:** 2022-10-27

**Authors:** Pallavi Harjpal, Moh'd Irshad Qureshi, Rakesh K Kovela, Moli Jain

**Affiliations:** 1 Physiotherapy, Ravi Nair Physiotherapy College, Datta Meghe Institute of Medical Sciences, Wardha, IND; 2 Neuro Physiotherapy, Ravi Nair Physiotherapy College, Datta Meghe Institute of Medical Sciences, Wardha, IND; 3 Physiotherapy, Nitte Institute of Physiotherapy, Nitte (Deemed to be University), Mangaluru, IND

**Keywords:** rehabilitation, stroke, walking, balance, unilateral training, bilateral training

## Abstract

Background and objective

While designing the rehabilitation regime of a hemiplegic patient, most physiotherapists focus on the affected/hemiparetic side. The less affected/unaffected side remains unused and loses its properties, i.e., muscle strength, girth, balance, and gait, thus causing deconditioning effects in patients’ overall rehabilitation. To enhance the recovery process, the focus should be drawn to training both sides equally to fasten the recovery process. The rationale behind designing this study was to maintain the integrity of the unaffected side along with rehabilitation of the affected side in hemiplegic patients. Many proven studies focus on bimanual upper-limb training in post-stroke survivors, but there is a lack of literature regarding the same in the lower limbs. This clinical trial was designed to study the effect of bilateral lower-limb training over unilateral lower-limb training on balance and walking in post-stroke survivors.

Methods

40 hemiplegic patients were selected and randomly divided into two groups: Group A (unilateral training group (UTG)) and Group B (bilateral training group (BTG)). Patients in Group A underwent approach-oriented training using the motor relearning program (MRP) and proprioceptive neuromuscular facilitation (PNF) for the affected side, while those in Group B underwent strength training for the lower-limb muscles using DeLorme's principle for the unaffected side and approach-oriented training using the MRP and PNF for the affected side for a period of six weeks, five days per week. A strengthening regimen was designed for the unaffected side, considering the frequency, intensity, time, and type (FITTs) principle provided by the American College of Sports Medicine (ACSM). The static and dynamic balance along with gait parameters were measured using the functional reach test (FRT), one-leg stance test (OLST), Berg balance scale (BBS), Dynamic Gait Index (DGI), gait parameters (stride length, gait velocity, and cadence), and Brunnstrom recovery stages (BRS) at the baseline and post rehabilitation.

Results

Both groups significantly improved following therapy (p<0.05). Group B showed more significant results both statistically and clinically. The enhancement in the FRT (2.25, p<0.03), OLST (5.12. p<0.0001), BBS (0.68, p<0.020), and DGI (1.70, p<0.030) scores indicated improvement in static and dynamic balance in the two groups. Patients showed improvement in the stereotyped sequence of movements indicating recovery on the BRS (4.62, p<0.0001). The overall gait parameters in patients, i.e., gait velocity (6.78, p<0.0001), stride length (3.59, p<0.001) and cadence (6.15, p<0.0001), improved post rehabilitation.

Conclusion

The results of this study showed that the BTG had positive impacts on the postural balance and walking capacities of subacute hemiparetic stroke patients, promoting early recovery in comparison to the UTG. This study also helped to design a strengthening protocol for the unaffected side according to DeLorme’s principle in line with the FITTs principle.

## Introduction

According to the WHO, stroke is the "incoming epidemic of the twenty-first century," defined as "the rapidly expanding clinical evidence of a permanent (or global) impairment of brain function, with symptoms lasting 24 hours or more or leading to death, for no apparent reason other than the vascular origin" [1,2]. In low- and middle-income nations, stroke accounts for 70% of all cases, causes 87% of fatalities, and reduces life expectancy through disability. Modified stroke prevalence rates in rural India range from 84 to 262 per 100,000 people, whereas rates in urban areas range from 334 to 424 per 100,000 people [3].

Based on etiology, stroke is categorized into two disparate variants: ischemic and hemorrhagic. An ischemic variant of stroke evolves whenever the vascularity of the tissues of the brain gets jeopardized as a result of any inherent reason, and it is encountered in a majority of stroke sufferers [4]. However, a hemorrhagic subtype of stroke dawns whenever an artery serving the blood to the brain is disrupted [5]. A large percentage of stroke victims experience hemiplegia (paralysis) or hemiparesis (weakness) on the contralateral side [6]. Aside from this, considerable weakness can be seen on the "seemingly normal" ipsilateral side of the lesion [7,8]. This is because only 75 to 90% of corticospinal fibers cross to the medulla's contralateral side. The remainder transmit ipsilaterally to the spinal cord by the anterior or ventral corticospinal tract. In the spinal cord, some of these fibers cross, while the remainder remain uncrossed, resulting in bilateral paralysis [9].

One of the most prevalent impairments in persons who have had a stroke is a loss of muscle strength. Muscle weakness leads to activity limitations i.e., walking, sit-to-stand transfers, stair climbing, and upper-limb and lower-limb activities. Strengthening of weak muscles using progressive resistive strength training (PRT) has evidence of positive effects in post-stroke survivors using the DeLorme and Watkins principles where resistance should be gradually raised [10]. The frequency, intensity, time, and type (FITT) principle is provided by the American College of Sports Medicine (ACSM) for stroke patients for strengthening the hemiplegic side, but there is no such consideration for the unaffected side, even though there is enough documentation that post-stroke survivors motor functioning is compromised on the unaffected side, as evidenced by muscular weakness, atrophy, or disuse [11-13].

Combining a bilateral training program with unilateral task-oriented training may be more beneficial for improving arm and hand function in those with chronic paresis than unilateral task-oriented training alone [14]. Bilateral arm training with auditory cueing enhances the functional and motor performance of the affected extremity in stroke survivors [15]. Exercises to strengthen the lower limbs on both sides may assist stroke survivors with getting out of a chair, climbing stairs, walking, and generally improving their quality of life [16]. There is not enough evidence in literature to support whether format, dosage, and strategy are best for enhancing static and dynamic balance as well as the spatial and temporal gait parameters. This dearth in literature is the main purpose of our study by using specific DeLorme’s progressive resistance training on the unaffected side, along with approach-oriented specific training on the affected side. Bilateral training patients are given the task-oriented technique, with the less affected side receiving strength training and the affected side receiving a motor relearning program (MRP) and proprioceptive neuromuscular facilitation (PNF).

Although it is known that the unaffected side begins to lose strength five days after a stroke, limited study has been done on the effects of bilateral lower limb strengthening training, and there is no prescribed strengthening protocol for that side [12]. As a result, both paretic and non-paretic lower limbs were trained in this study, and a fixed regimen for the unaffected side training was designed and implemented.

## Materials and methods

Participants

**Figure 1:**
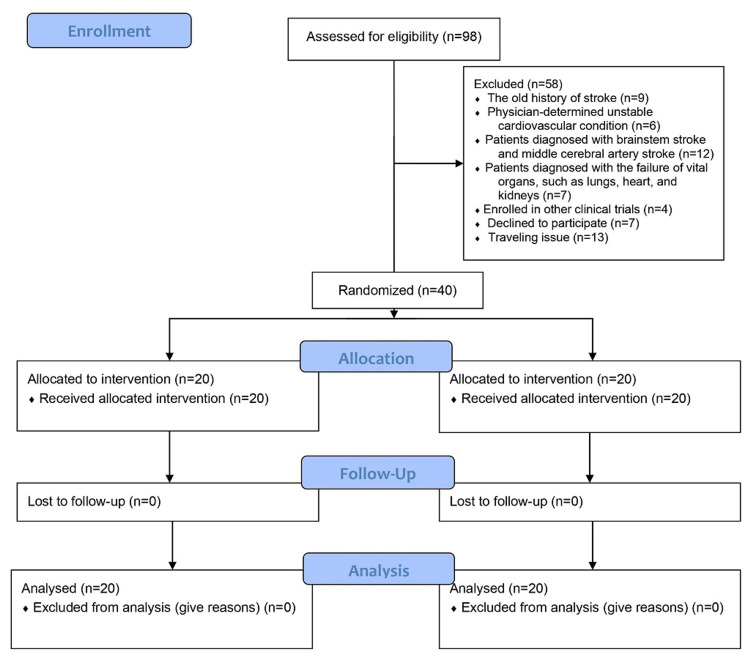
CONSORT flow chart of the study procedure CONSORT: Consolidated Standards for the Reporting of Trials

The study included people who had experienced their first stroke and were living in the community between the ages of 45 and 65. This age range was selected to achieve homogeneity and to take into account the age range during which strokes are most common [3]. G. Power 3.1.9.4 program used information from Banerjee and Das (2016) to calculate the number of study participants [3]. The study included 40 patients (20 each in both Groups A and B). The hiring process was placed in a straight line from June 2021 to June 2022. 98 people in total were examined for eligibility. The conduct and reporting of the trial were standardized using Consolidated Standards for the Reporting of Trials (CONSORT) [17]. The inclusion criteria were that the participants had to be of either gender between the age group of 40 to 65 years age group, in the sub-acute stage (one week to six months) of stroke, be able to understand and follow instructions, be willing to participate in the study, and be able to complete the outcome measures. Patients with a history of an old stroke (stroke with no active findings on radiological imaging), transient ischemic attack, or recurrent stroke; patients with any unstable cardiovascular condition, as determined by the physician; patients diagnosed with brainstem stroke and middle cerebral artery stroke; patients diagnosed with the failure of vital organs such as lungs, heart, and kidneys; and those who are currently enrolled in another research trial were excluded from the study. All the participants provided written informed consent. 40 participants were randomized in total, and baseline information is available. Figure 1 shows a flowchart of the study's methodology.

Study design

A randomized clinical trial was performed at the neuro-physiotherapy outpatient department of Ravi Nair Physiotherapy College and Acharya Vinoba Bhave Rural Hospital, Sawangi, Meghe, Wardha, India. Approval was taken from the Committee for Institutional Ethical Committee of the Datta Medical Science, Datta Meghe Institute of Medical Sciences (DMIMS, DU), Sawangi, Meghe, Wardha, India (Ethical permission number: DMIMS[DU]/IEC/2021/248) and Clinical Trial Registration of India (CTRI/2021/05/033621). The included participants diagnosed with subacute stroke were randomized through simple random sampling and allocated through the sequentially numbered opaque sealed envelope (SNOSE) method into Group A (unilateral training group (UTG)) and Group B (bilateral training group (BTG)). Randomization and allocation were done by the primary researcher who is a postgraduate resident in physiotherapy under the guidance of a professor of the neuro-physiotherapy department. Outcomes were assessed before the beginning of the study and immediately after the completion of the study by a postgraduate resident in physiotherapy of the same experience, who was aware of the study and blinded about the intervention. All aspects of the study's enrollment, intervention, and evaluation plans adhered to the established protocol requirements: a suggestion for conducting intervention trials [18].

Intervention

40 hemiplegic patients were selected and randomly divided into two groups: Group A (UTG) and Group B (BTG). Patients in Group A received approach-oriented training for the affected side using MRP and PNF, and Group B received strength training for muscles of the lower limb using DeLorme’s principle for the unaffected side and approach-oriented training for the affected side using MRP and PNF for five days/week for six weeks.

**Figure 2:**
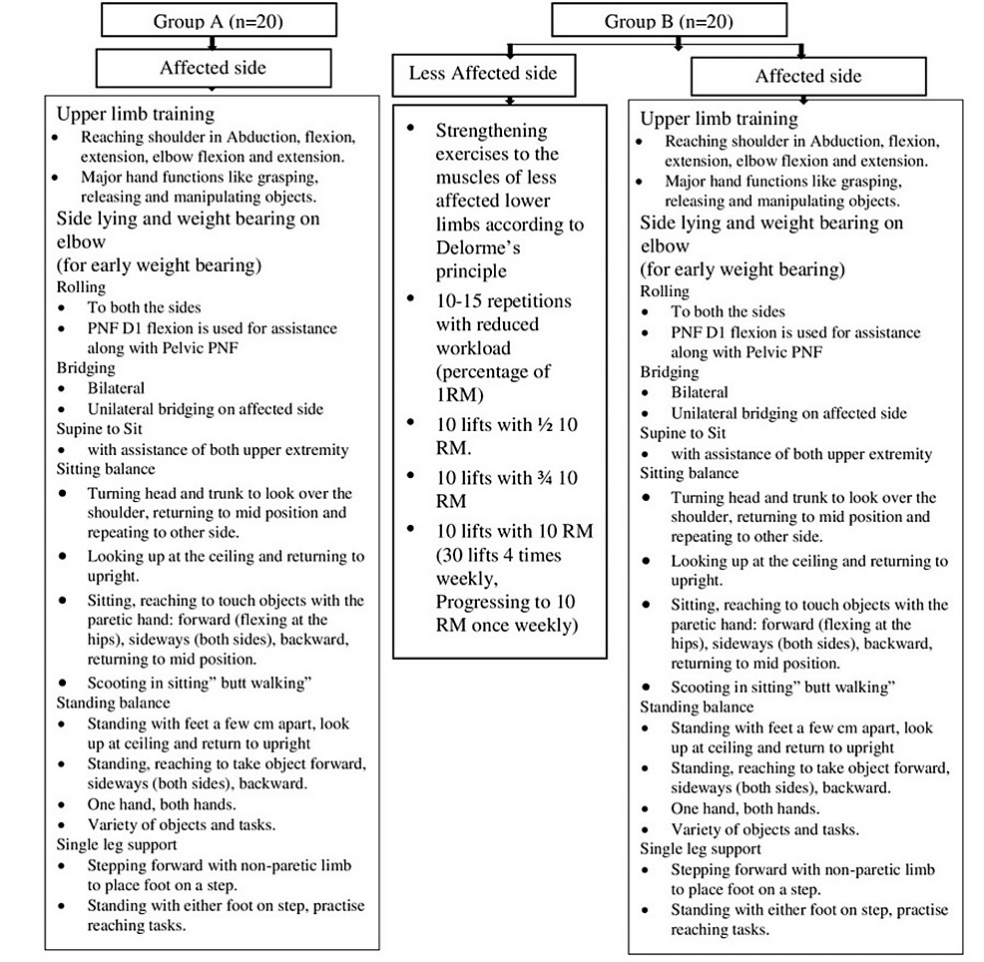
Intervention provided to the patients in both the groups The MRP, PNF, DeLorme's strengthening principle, and FITT principles were used to design the rehabilitation [6,11,19]. PNF: Proprioceptive neuromuscular facilitation; RM: Repetition maximum; MRP: Motor relearning program; FITT: Frequency, intensity, time, and type

Group A:For five days a week for a period of six weeks, the participants in this group underwent 20 minutes of lower limb training on the affected side only by a physiotherapist. The MRP and PNF (Figure 2) were both part of it, task-specific training and multiplanar motions of the affected lower and upper extremities are included [19].

Group B:This group got bilateral lower-limb training that comprised a task-oriented strategy for the damaged side, such as the MRP and PNF, as well as strengthening of the less affected side [20,21]. A physiotherapist prescribed strength training for 20 minutes on the side that was less affected, as well as 20 minutes of lower- and upper-limb exercises for the involved side five days a week for six weeks. The hip flexors, abductors and extensors, knee extensors, and ankle dorsiflexors were stengthened [12]. Each muscle was provided strength training for three minutes with a rest period of one minute in between each muscle group. Before training, each muscle group was tested for one repetition maximum (RM) using DeLorme’s boot. Post measurement, the strengthening was provided via a weight cuff tied over the ankle according to the strengthening regimen prescribed by DeLorme. The whole intervention design is displayed in Figure 2 [6,11,19]. 

Primary outcome measures

The primary outcome measures included balance post intervention. The functional reach test (FRT) is a clinical outcome test that assesses complicated balance in a single task. By measuring the patient's maximum forward reach while standing in a fixed posture, the FRT assesses a patient's stability. One-leg stance test (OLST) or single-leg stance test is a tool that is used to evaluate static posture and balance control. This technique is performed with eyes open and hands on hips. A client is more likely to fall and get an injury if they cannot stand for more than five seconds. Gait parameters, i.e., stride length, cadence, and gait velocity was assessed by 10-meter walk test. Berg balance scale (BBS) is a clinical evaluation that is frequently employed to assess a subject's static and dynamic balance. It is largely acknowledged as the best tool available for actual balancing tests. The 14 basic balance tasks in this evaluation, which take between 15 and 20 minutes to complete, range from standing to one-foot standing while seated. Dynamic Gait Index (DGI) is a tool for evaluating gait, balance, and the risk of falling that has been shown in studies. This requires both frequent steady-state walking and walking while engaging in more demanding activities.

Secondary outcome measures

The Brunnstrom recovery stages (BRS) is a performance-based outcome that is based on the stereotyped sequence of events that take place during the recovery process from a stroke. It has separate components for the upper limb, trunk, and lower limb. For lower-limb evaluation, the patient is tested supine, then sitting, then standing, and if possible, gait is evaluated. The return of function is stated from Stage 1 to Stage 6. The patient’s stage of recovery from the time of the episode helps in early prognosis.

Statistical analysis

Descriptive and inferential statistics were carried out using the chi-square test, Student's paired and unpaired t-tests, and software from SPSS 27.0 (IBM SPSS, Armonk, USA) and GraphPad Prism 7.0 (GraphPad Software, San Diego, USA). The level of significance for the statistical analysis was established at p<0.05. Student’s paired t-tests were used to find the statistical difference between Groups A and B, and it came out to be significant.

## Results

In all, 98 patients were enrolled, and eligibility was determined for each. 58 of those patients were left out: 26 people refused to participate, 19 did not meet the requirements for participation, and 13 had additional justifications. The study's 40 patients who met the inclusion requirements formed Group A and Group B. Patients in Group A were 40-63 years old on average, whereas those in Group B were 40-65 years old, with a gender distribution of 12:8 and 11:9 (men: women), respectively. Using the chi-square test, it was determined that there was no statistically significant difference between the two groups' patient ages (p-value=0.91). The baseline characteristics of the subjects are described in Table 1. The patients with the anterior cerebral artery stroke in the subacute phase, i.e., a week to six months from the stroke were in the inclusion criteria.

**Table 1:** Baseline characteristics NS: Not significant

Baseline Characteristics	Group A	Group B	p-value
Age in years	51.75±7.06	51.50±8.40	0.91, NS
Age Range	40-63 yrs	40-64 yrs	
Gender			
Male	12 (60%)	11 (55%)	0.54, NS
Female	8 (40%)	9 (45%)	

Table 2 indicates the statistical analysis of the measured outcome measures as well as the significant value post rehabilitation between groups. This study found that bilateral lower-limb strengthening exercise benefited hemiparetic stroke patients regain their balance, i.e., Group B showed a larger improvement in outcome measure scores than Group A.

**Table 2:** Mean FRT, OLST, gait velocity, stride length, cadence, BBS, DGI, and BRS pre and post treatment of Groups A and B and inter-group analysis FRT: Functional reach test; OLST: One-leg stance test; BBS: Berg balance scale; DGI: Dynamic Gait Index; BRS: Brunnstrom recovery stages

Outcome measure	Group A		p-value	Group B		p-value	Mean Difference (X±SD)		p-value
	Pre-treatment	Post-treatment		Pre-treatment	Post-treatment		Group A	Group B	
FRT	11.55±1.84	28.20±3.56	0.0001	12.65±2.13	32±3.74	0.0001	16.65±4.14	19.35±3.39	0.030
OLST	0.85±0.36	4.40±0.59	0.0001	0.90±0.44	5.60±0.75	0.0001	3.55±0.60	4.70±0.80	0.0001
Gait velocity (m/sec)	0.03±0.01	0.21±0.05	0.0001	0.04±0.01	0.35±0.07	0.0001	0.17±0.05	0.31±0.07	0.0001
Stride length (cm)	19±2.80	56.40±8.50	0.0001	21.60±3.58	68.85±7.12	0.0001	37.40±9.27	47.25±7.98	0.001
Cadence (steps/min)	24.60±3.16	46.30±5.33	0.0001	26.15±3.95	62.65±9.17	0.0001	21.70±6.39	36.50±8.65	0.0001
BBS	23.85±3.99	39.45±4.68	0.0001	29.80±6.62	46.60±3.05	0.0001	13.60±3.63	16.80±6.92	0.020
Dynamic Gait Index	7.65±1.53	17.30±2	0.0001	8.55±1.98	19.30±1.71	0.0001	9.65±2.05	10.75±2.02	0.030
BRS	2.50±0.51	4.05±0.75	0.0001	2.45±0.51	5.10±0.64	0.0001	1.55±0.75	2.65±0.74	0.0001

**Figure 3:**
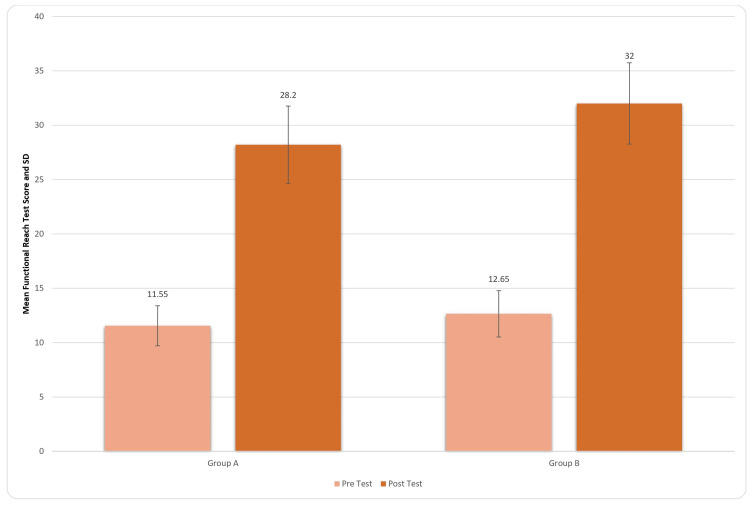
Comparison of FRT scores in two groups pre and post treatment FRT: Functional reach test

The Figure 3 depicts the pre- and post-treatment scores of the FRT. There was an improvement in both the groups post treatment, but Group B shows more significant results. For inter-group analysis, Student’s unpaired t-test was used, and the results came out to be significant (t-value=2.25, p-value=0.030) for FRT. This indicates an improvement in the dynamic balance of the patients.

**Figure 4:**
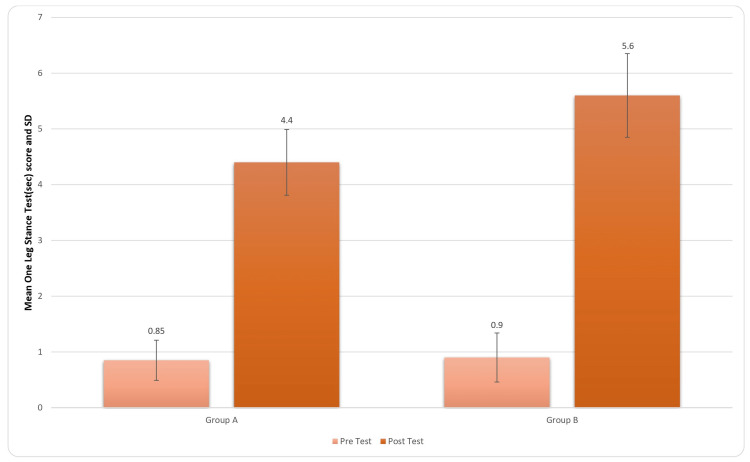
Comparison of OLST (sec) score in two groups pre and post treatment OLST: One-leg stance test

Similarly, in Figure 4, the representation of pre- and post-treatment OLST scores is provided. There were commendable results in Group B, indicating improvement in the static balance of the patient post treatment. For inter-group analysis, Student’s unpaired t-test was used, and the results came out to be significant (t-value=5.12, p-value=0.0001) for OLST.

**Figure 5:**
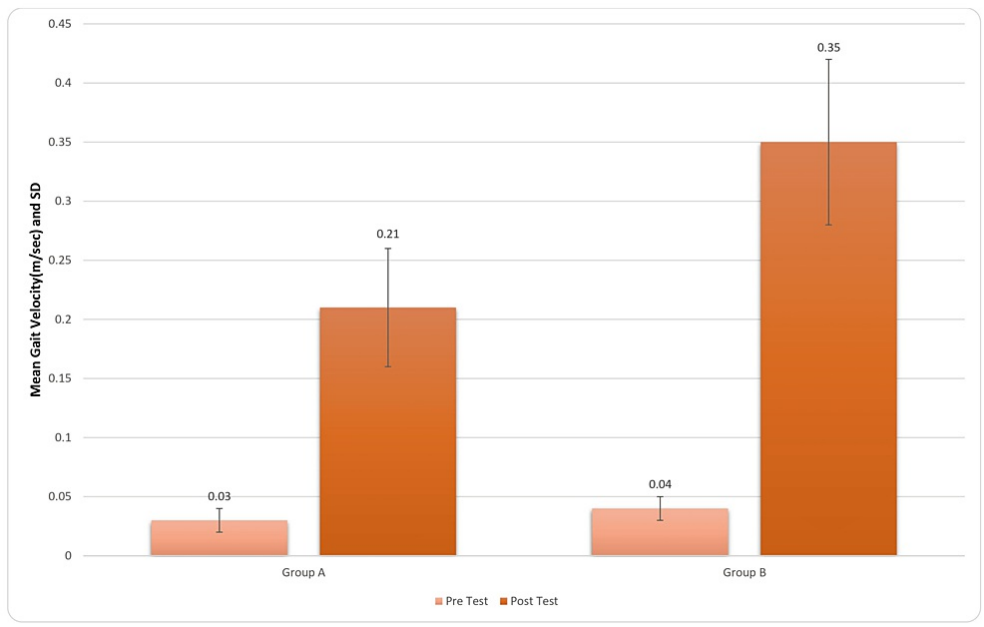
Comparison of gait velocity (m/sec) score in two groups pre and post treatment

Figure 5 provides the results in gait velocity which were great both clinically and statistically. For inter-group analysis, Student’s unpaired t-test was used, and the results came out to be significant (t-value=6.78, p-value=0.0001) for gait velocity.

**Figure 6:**
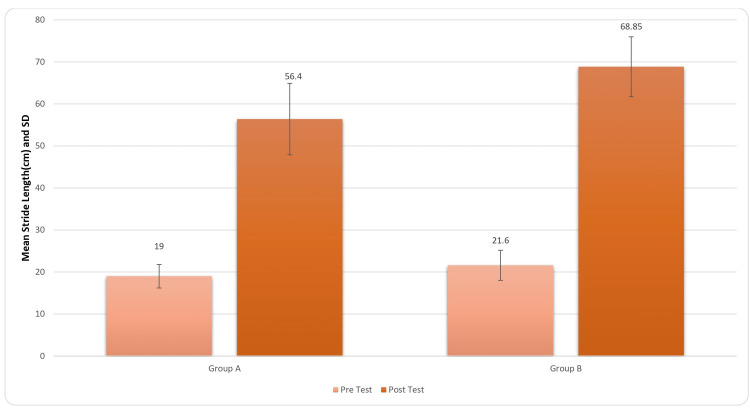
Comparison of stride length (cm) in two groups pre and post treatment

Figure 6 provides the results in the improvement of stride length of the patient post rehabilitation. For inter-group analysis, Student’s unpaired t-test was used, and the results came out to be significant (t-value=3.59, p-value=0.0001) for stride length.

**Figure 7:**
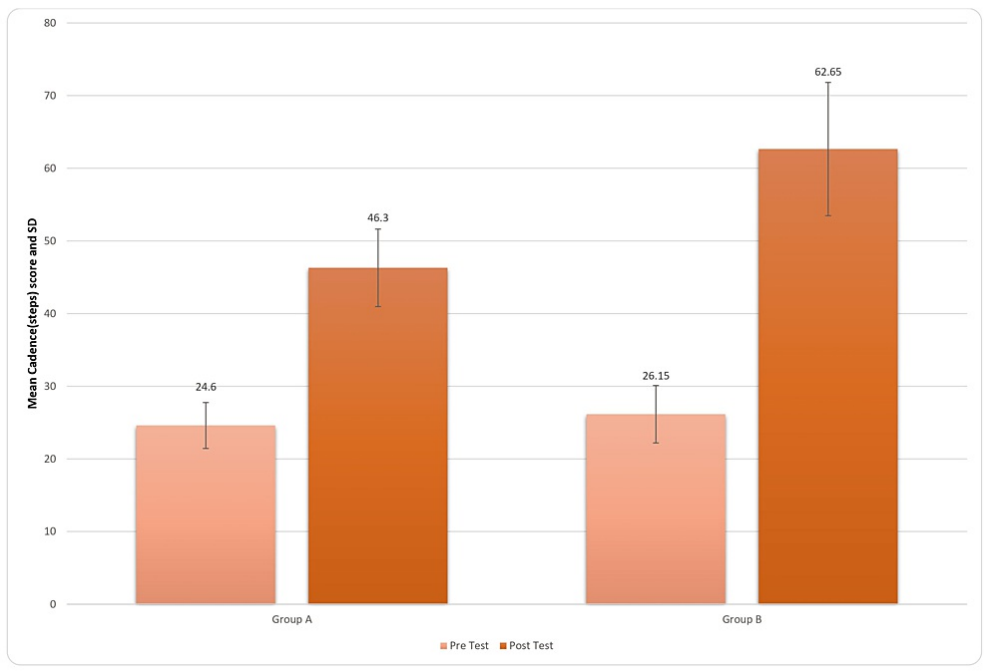
Comparison of cadence (steps) in two groups pre and post treatment

Figure 7 provides the positive effects of the treatment protocol of the patients post rehabilitation on the steps/min, i.e., cadence. This suggested that the treatment provided also enhances gait parameters. For inter-group analysis, Student’s unpaired t-test was used, and the results came out to be significant (t-value=6.15, p-value=0.0001) for cadence score.

**Figure 8:**
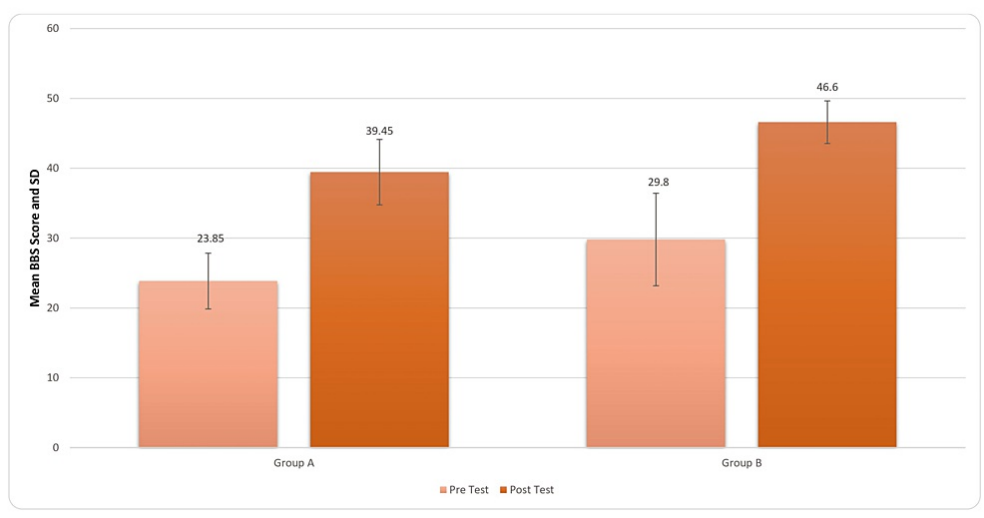
Comparison of total BBS score in two groups pre and post treatment BBS: Berg balance scale

There was an improvement in the patients' balance post rehabilitation. For inter-group analysis, Student’s unpaired t-test was used, and the results came out to be significant (t-value=0.68, p-value=0.020) for the BBS score. It is indicated in Figure 8.

**Figure 9:**
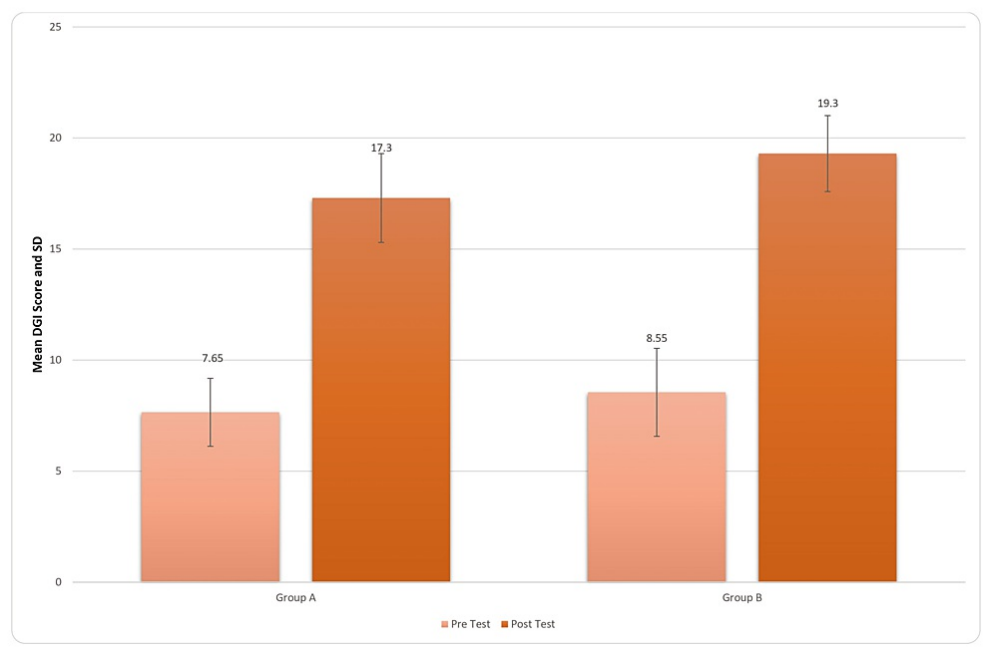
Comparison of DGI scores in two groups pre and post treatment DGI: Dynamic Gait Index

For inter-group analysis, Student’s unpaired t-test was used, and the results came out to be significant (t-value=1.70, p-value=0.030) for the DGI score indicated in Figure 9.

**Figure 10:**
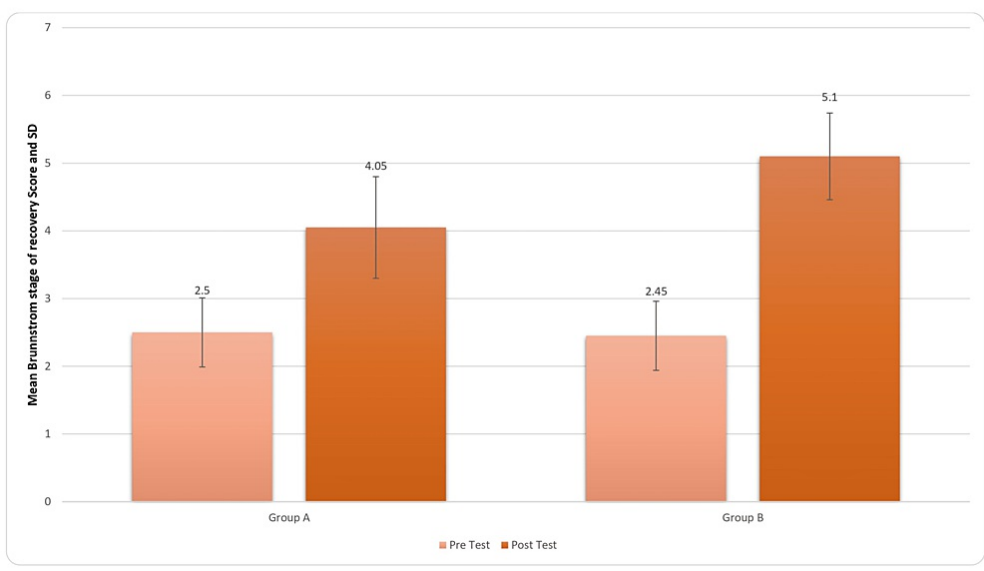
Comparison of BRS Score in two groups pre and post treatment BRS: Brunnstrom recovery stages

There was refinement in the movement pattern from the stereotyped movement pattern indicated by BRS (see Figure 10). For inter-group analysis, Student’s unpaired t-test was used, and the results came out to be significant (t-value=4.62, p-value=0.0001) for the BRS score.

## Discussion

The goal of stroke rehabilitation is to enable individuals who have had a stroke to return to having as close to a normal life as possible by reclaiming their daily skills. Different therapeutic options have been used to help people who have suffered from a stroke to improve their quality of life. While developing the rehabilitation regimen for a hemiplegic patient, most physiotherapists focus on the affected/hemiparetic side. The less affected/not affected side stays unused and loses its properties, thus causing deconditioning effects in the overall rehabilitation of patients. To enhance the recovery process, emphasis should be placed on training both. The rationale for this study was to maintain the integrity of the unaffected side as well as the rehabilitate the impacted side in hemiplegic patients.

We believe this study is the first to document the key benefits of strengthening the unaffected lower limb to enhance balance and gait in stroke survivors. Strength impairment on the unaffected side was stated in 1995 when individuals with stroke had muscle strength impairment which was more proximally than distally for both upper limbs and lower limbs. These correlations provide support for the inclusion of bilateral strength training activities in the management of post-stroke survivors [12]. Static balance and proprioception are instantly impacted by strengthening training as an intervention strategy in healthy adults. It is suggested that strengthening exercises for the gluteus medius should be targeted since the group of subjects who received strengthening training in their weak areas fared substantially better than the group who received relaxation therapy in their strong areas [22].

In a systemic review of a similar nature on the upper limb in 2020, it was found that training both arms may be more helpful than training only one in hastening the recovery of upper-limb function after stroke [23]. In a similar study in 2021, six weeks of bilateral training illustrated a drastic improvement showing the importance of training both sides in patients with hemiplegia [24]. A study in 2018 showed the positive effects of bilateral lower limb strengthening on the balance and walking in hemiplegic patients. It also showed that the non-paretic limb strength also a has significant effect on balance [25].

The FRT was 32±3.74 cm in all of the participants of Group B while in Group A, it was 28.20±3.56. Recurrent falls were linked to a score of 17.78 cm in a previous study, which has been utilized to look into the effects of balance and falls in patients after stroke suggesting an improvement in dynamic balance [26,27]. The OLST, which is used to assess static posture and balance control, provided a significant result in our study. As the suggested normative value indicates, the inability to stand for less than five seconds suggested a greater risk of injury from a fall. Most of our subjects approached the normative value post six weeks of intervention. Walking speed, which is frequently decreased in stroke patients, is crucial for evaluating gait. In healthy people, the average walking speed is 1.3 m/s, whereas, in people with hemiplegia, it ranges from 0.23-0.73 m/s [28,29]. Both groups improved their gait velocity as a result of our research, with Group B showing a mean of 0.35, indicating normative values for hemiplegic patients. The majority of the patients in both groups showed improvement in stride length. The mean came out to be 68.85 cm. Bytyci and Henein stated that a stride length of 64 cm accurately predicts the occurrence of future clinical issues, suggesting that it could be used to guide individuals to the best possible exercise and assistance [30]. In this clinical trial, 17 participants in Group B and 11 in Group A achieved more than 64 cm stride length. Similar effects were found in cadence, suggesting an improvement in overall gait. The improvement in stride length can be correlated with the improvement in cadence [30]. It is in line with previous research, which states that in addition to traditional therapy after a stroke, bilateral isokinetic strengthening exercise appears to be useful in strengthening muscles on both sides and enhancing gait, balance, and functional characteristics [31].

The majority of the patients (34 out of 40) in the current study complained of a lack of balance. The BBS is a recognized tool to measure the static and dynamic balance in stroke patients. The mean BBS score for all of the participants of Group B was 46.60±3.05, indicating that most of them (17 out of 20) were at minimal risk of falling as, according to previous studies, scores of 45 have been linked to an increased risk of falling [32,33]. In an original article, it is stated that the most effective treatment for increasing strength seems to be progressive resistance training [34]. It greatly boosts strength when it is properly targeted, thereby improving the balance and reducing the gait deviations [34,35]. The BRS in our subjects also improved post treatment, which is also found out in similar studies [36].

In our study, FRT, OLST, gait parameters, BBS, DGI, and BRS of the lower limb showed more improvement in the BTG post rehabilitation. Training both sides aided in both early recovery and the creation of a foundation for future studies on how emphasizing the unaffected side can enhance the capabilities of the affected side. As a result, physiotherapists should use bilateral lower-limb training to efficiently restore balance and rehabilitate gait in post-stroke survivors to achieve the highest level of self-independence.

Limitations and future scope

It is important to recognize the study's several shortcomings. As this was a time-bound study, follow up was not possible, suggesting the long-term effects were not assessed. Sub-acute stroke survivors were included. Thus, generalization of results was not possible. This study's randomized controlled design and standardized training regimen with pre-established exercises are its greatest strengths. All of the evaluations were carried out by the same independent physiotherapist, which lessened the risk to internal validity. The narrow age range can be seen as a strength because a bigger age range would probably have resulted in considerably greater variance in the outcomes.

The results of this study demonstrate the benefits of strengthening the unaffected side combined with approach-oriented training for the affected side in post-stroke survivors. A similar study with different patients in acute and chronic stages of stroke can be done in the future. This study also helped to design a set strengthening regimen for the unaffected side of hemiparetic patients, further enabling many new types of research.

## Conclusions

Reduction in muscle strength post stroke is one of the causes of delayed recovery. In this randomized clinical trial, we found that both groups improved in terms of static and dynamic balance, gait parameters, and overall stroke-specific index of disability. When both groups were compared, the BTG showed more significant results clinically and statistically on comparison of all the outcome measures, i.e., FRT, OLST, gait parameters, BBS, DGI, and BRS of the lower limb. Training both sides not only helped in early recovery but also helped to establish a base for further research on how focusing on the unaffected side helps to improve the competencies of the affected side. As a result, physiotherapists should use bilateral lower-limb training to efficiently restore balance and rehabilitate gait in post-stroke survivors to achieve the highest level of independence.
